# Self-Stigma in Parkinson's Disease: A 3-Year Prospective Cohort Study

**DOI:** 10.3389/fnagi.2022.790897

**Published:** 2022-02-11

**Authors:** Junyu Lin, Ruwei Ou, Qianqian Wei, Bei Cao, Chunyu Li, Yanbing Hou, Lingyu Zhang, Kuncheng Liu, Huifang Shang

**Affiliations:** Laboratory of Neurodegenerative Disorders, Department of Neurology, Rare Diseases Center, West China Hospital, Sichuan University, Chengdu, China

**Keywords:** Parkinson's disease, self-stigma, depression, cohort study, epidemiology

## Abstract

**Purpose:**

Self-stigma is common in patients with Parkinson's disease (PD) and may lead to social isolation and delayed search for medical help. We conducted a 3-year prospective longitudinal study to investigate the development and evolution of self-stigma in patients with early stage PD and to explore the associated and predictive factors of self-stigma in PD.

**Method:**

A total of 224 patients with early stage PD (disease duration <3 years) were enrolled at baseline and followed up annually for 3 consecutive years. Self-stigma was assessed by the stigma subscale of the Parkinson's Disease Questionnaire (items 23–26). The generalized estimating equation model was used to investigate the associated factors of self-stigma over 3 years, and the binary logistic model was used to explore the predictors of self-stigma in patients with PD without self-stigma at baseline.

**Results:**

The prevalence of self-stigma decreased from 58.0% at baseline to 49.2% after 3 years. The Hamilton Depression Rating Scale (HDRS) score was the only associated factor [B: 0.160 (1.106–0.214), *P* < 0.001] of self-stigma over 3 years and the only predictor [OR: 1.252 (1.044–1.502), *P* = 0.015] of the onset of self-stigma.

**Conclusion:**

Self-stigma is very common in PD, but its prevalence tends to decrease as the disease progresses. Depression was the only associated and predictive factor of self-stigma in PD and could be an effective target of alleviating self-stigma.

## Introduction

Parkinson's disease (PD) is the second most common neurodegenerative disorder characterized by a variety of motor and non-motor symptoms such as bradykinesia, resting tremor, stiffness, postural instability, constipation, hyposmia, depression, sleep problems, cognitive impairment, and so on (Poewe et al., 2017). As a chronic neuropsychiatric disease, PD is often accompanied by stigma (Hermanns, 2013; Ma et al., 2016). Stigma comprises social stigma and self-stigma. Social stigma refers to negative stereotypes from the society, which can lead to prejudice and discrimination. Once the negative stereotypes are perceived by the patient, self-stigma occurs, which is usually associated with decreased self-esteem and self-efficacy (Molina et al., 2013). A study found that about half of the patients with PD had experienced self-stigma (Ma et al., 2016). Self-stigma in neuropsychiatric diseases may lead to harmful consequences, such as social isolation, delayed search for medical help, non-adherence to treatment, and increased suicide rates (Corrigan, 2004; Schomerus et al., 2015). Previous studies have shown that self-stigma is a key determinant of health-related quality of life in PD (Ma et al., 2016), and it is associated with difficulties in activities of daily living (ADL) (da Silva et al., 2020) and high burden for caregivers (Tan et al., 2019) in patients with PD. Thus, studying the determinants of self-stigma in PD might help to intervene and reduce it.

A few cross-sectional studies have explored the factors associated with self-stigma in patients with PD, which yielded varied findings. A study found that age and depression were associated with self-stigma in male patients with PD, while depression was the only factor associated with self-stigma in female patients with PD (Salazar et al., 2019). Another study has found that age, non-motor symptoms, Hoehn & Yahr (H&Y) stage, and marital status were associated with self-stigma in patients with PD (Wu et al., 2014). One study has found that apathy and depression were associated with self-stigma in patients with PD (Oguru et al., 2010). Another study has focused on the impact of motor complications found that biphasic dyskinesia, morning akinesia, end-of-dose fluctuations, and unpredictable offs were associated with self-stigma in patients with PD (Chapuis et al., 2005). Up to date, no longitudinal study has been conducted to investigate the evolution and determinants of self-stigma in PD. Therefore, the aims of this study were to identify the prevalence, evolution, associated factors, and risk factors of self-stigma in patients with PD from a 3-year prospective longitudinal PD cohort.

## Materials and Methods

### Evaluation of Patients

This study was approved by the Ethics Committee of West China Hospital of Sichuan University, and all participants have signed an informed consent. We reported a 3-year prospective longitudinal cohort study according to the Strengthening the Reporting of Observational Studies in Epidemiology (STROBE) reporting guideline for cohort studies. All patients were consecutively selected from the Department of Neurology, West China Hospital of Sichuan University, between February 2014 and May 2016. The inclusion criteria were listed as follows: (1) patients who met the United Kingdom Brain Bank diagnostic criteria of PD (Hughes et al., 1992); (2) patients with early PD (disease duration <3 years); and (3) patients who were of Han nationality. The exclusion criteria were listed as follows: (1) patients who refused the annual follow-up; (2) patients who could not complete a face-to-face interview; and (3) patients whose educational years were <3. A total of 224 patients with PD were finally included. The sample size and power were sufficient according to a previous report (Liu and Liang, 1997).

We collected demographic and clinical data of the participants at baseline, including sex, age, age of onset, disease duration, and educational years. All the participants underwent a face-to-face interview by trained movement disorder specialists at baseline and every 12 months during the following three consecutive years of follow-up. Work status, marriage status, and medicine use, including antiparkinsonian and antidepressant drugs, were collected at baseline and at each visit during follow-up. Levodopa equivalent daily doses (LEDD) was calculated by the commonly used protocol (Tomlinson et al., 2010). Motor and non-motor symptoms were repeatedly assessed using standard scales at each interview. The severity of parkinsonism was assessed using the Unified Parkinson's Disease Rating Scale Part III (UPDRS III) (Movement Disorder Society Task force on rating scales for Parkinson's, 2003). The ADL was assessed using the UPDRS II (Goetz et al., 2008). The disease stage was assessed using the H&Y scale (Hoehn and Yahr, 1967). The burden of non-motor symptoms was assessed using the Non-Motor Symptoms Scale (NMSS) (Wang et al., 2009). Depression and anxiety were screened using the Hamilton Depression Rating Scale-24 (HDRS-24) (Hamilton, 1967) and the Hamilton Anxiety Rating Scale (HARS) (Hamilton, 1959), respectively. The HDRS-24 score > 20 indicated depression (Gibbons et al., 2012). The Montreal Cognitive Assessment (MoCA) (Nasreddine et al., 2005) was used to evaluate the global cognitive function, and the Frontal Assessment Battery (FAB) (Dubois et al., 2000) was used to evaluate the executive function. The presence of fluctuation and dyskinesia were collected at each visit (Supplementary Table 1).

Self-stigma was assessed using the stigma subscale of the Parkinson's Disease Questionnaire (PDQ-39) (Jenkinson et al., 1997) items 23–26. Each item was scored 0–4, where 0 represents never and 4 represents always. The total self-stigma scores ranged from 0 to 16. The presence of self-stigma was defined as a total stigma score ≥ 1 of 16 for maximum sensitivity to screen out the patients with self-stigma.

### Statistical Analysis

The continuous variables were presented as mean and standard deviation (SD) if normally distributed and as the median and interquartile range (IQR) if non-normally distributed. All categorical variables were presented as numbers and percentages. Demographic and clinical characteristics of patients with or without self-stigma were compared at baseline and at 3-year follow-up using Student's *t*-test for normally distributed continuous variables, Mann-Whitney test for non-normally distributed continuous variables, and chi-squared test or Fisher's exact test for categorical variables. Baseline demographic and clinical characteristics were also compared between patients who completed the 3-year follow-up and those who did not. To avoid false-positive significances, the *p*-values were false discovery rate (FDR)-corrected for multiple comparisons following the Benjamini-Hochberg procedure (Feser et al., 2009).

The generalized estimating equation (GEE) model with the method of multiple linear regression, which allowed for correlation between repeated measurements of the same patients, was applied to investigate the associated factors of self-stigma over 3 years (Salazar et al., 2016). GEE is a method that can use all the available information, without excluding any individual even if they are missing at some time points. Therefore, our “monotone dropout” missing data can be handled exactly by the GEE model (Salazar et al., 2016). The dependent variable, the self-stigma score, was used as a continuous variable in this model. Independent fixed variables included sex, age at baseline, age of onset, disease duration at baseline, and educational years. Independent repeated variables included work status, marriage status, antidepressant drugs (yes/no), LEDD, sores of UPDRS III, UPDRS II, HARS, HDRS, NMSS, MOCA, and FAB, H&Y stage, fluctuation (yes/no), dyskinesia (yes/no), and follow-up time in years. An exchangeable working correlation structure was chosen. The GEE analyses were first conducted with only one variable evaluated at a time (unadjusted model). Then, variables with a *p-*value < 0.10 and those were thought clinically significant (such as sex, scores of HDRS, HARS, and UPDRS III) were included in the multivariate GEE analysis (adjusted model).

The binary logistic model was used to explore the baseline predictors for developing self-stigma (self-stigma score ≥ 1) in 3 years in PD. Patients without self-stigma (self-stigma score = 0) at baseline were included in the analysis. Potential baseline predictive variables, including sex, age, age of onset, disease duration, educational years, work status, marriage status, antidepressant drugs (yes/no), LEDD, scores of UPDRS III, UPDRS II, HARS, HDRS, NMSS, MOCA, and FAB, and H&Y stage, were evaluated first using a univariate logistic model. Then, candidates with a *p*-value < 0.10 and those were thought clinically significant (such as sex, scores of HDRS, HARS, and UPDRS III) were included in the multivariate logistic model (Harrell et al., 1988).

All analyses were performed using the Statistical Package for the Social Sciences (SPSS) version 22.0. Two-tailed *p-*values of < 0.05 were considered statistically significant. Data were analyzed from October 2020 to February 2021.

## Results

### Baseline and Follow-Up

**Figure 1:**
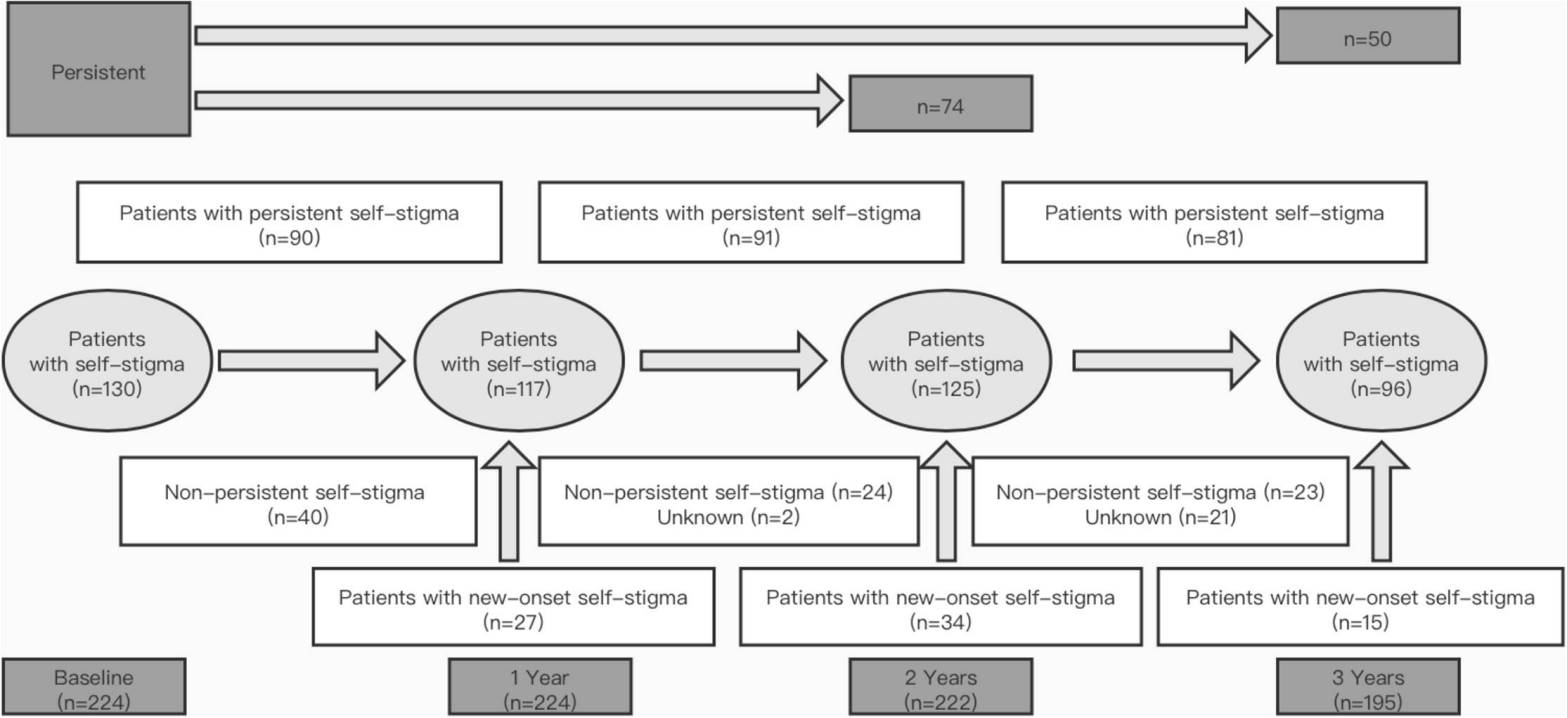
Prevalence and persistence of self-stigma in patients with Parkinson's disease **(PD)** over 3 years. “Patients with persistent self-stigma” means that self-stigma was persistent from one visit to the next; “Non-persistent self-stigma” means that self-stigma was present in a patient at the previous follow-up but absent at the next follow-up; “Patients with new-onset self-stigma” means that the self-stigma was absent in a patient with PD at the previous follow-up but present at the next follow-up.

A total of 224 Chinese patients with early stage PD (121 males) were included at baseline. The average age of the patients was 57.60 (SD: 11.10) at baseline, with a mean disease duration of 1.52 (SD: 0.86) years. All the 224 (100%) patients were available to be reassessed at 1 year; 222 (99.1%) patients were available at 2 years and 195 (87.1%) were available at 3 years (Figure 1). No significant difference in baseline characteristics was identified between the patients with and without completion of 3-year follow-up (Supplementary Table 2).

### Development and Evolution of Self-Stigma

**Figure 2:**
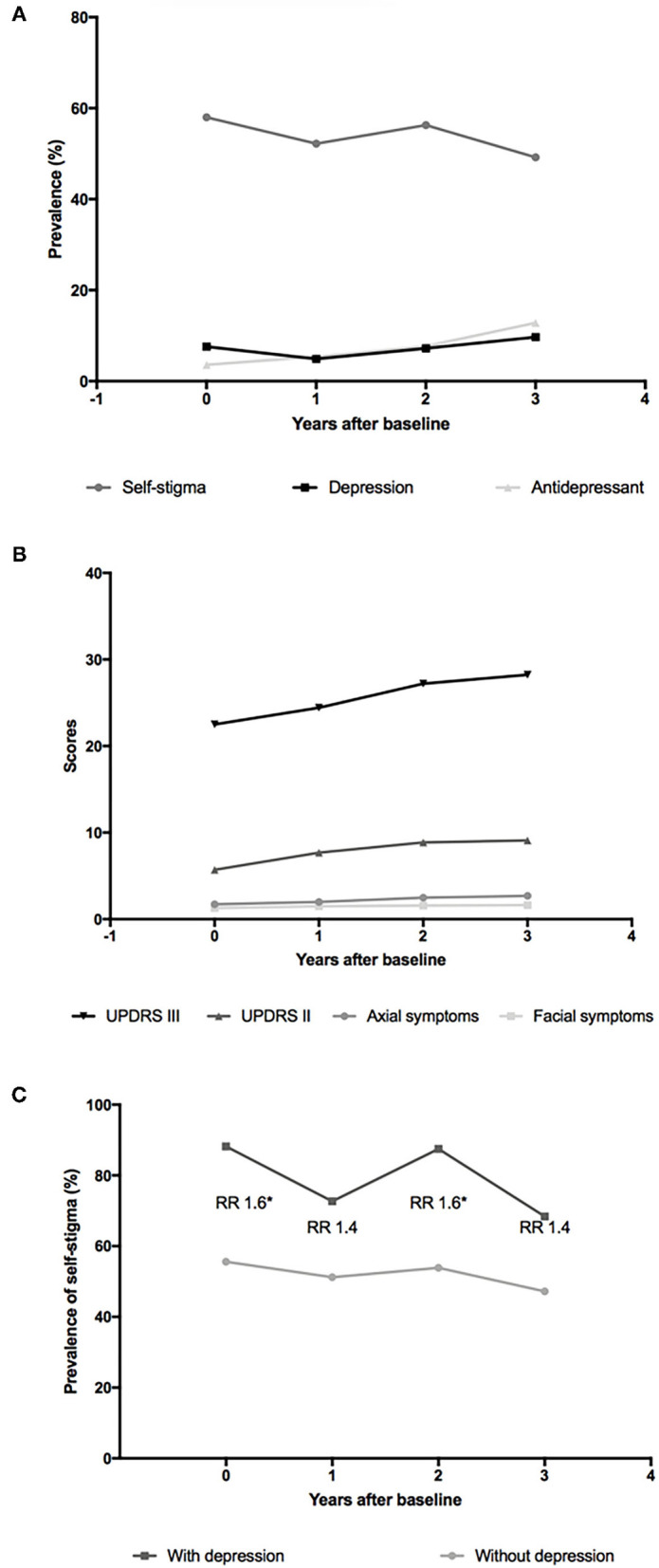
Evolution of self-stigma and other symptoms in patients with PD over 3 years. **(A)** Point prevalence of self-stigma, depression, and antidepressant use in patients with PD over 3 years; **(B)** Scores of Unified Parkinson's Disease Rating Scale Part II (UPDRS II), UPDRS III, axial symptoms, and facial symptoms in patients with PD over 3 years; **(C)** Point prevalence of self-stigma in patients with PD with and without depression over 3 years. The numbers of participants with depression were 17 at baseline and 11, 16, and 19 at 1-, 2-, and 3-year follow-ups. The numbers of participants without depression were 207 at baseline and 213, 206, and 176 at 1-, 2-, and 3-years follow-ups. RR, relative risk. *p < 0.05.

The development and evolution of self-stigma are shown in Figure 1. The prevalence of self-stigma in PD was 58.0% at baseline, 52.2% at 1 year, 56.3% at 2 years, and 49.2% at 3 years. Overall, although the prevalence of self-stigma did not reach a statistical significance (*p* = 0.071), it tended to decrease during the 3-year follow-up (Figure 2A). Self-stigma was not always persistent from one visit to the next in all patients during the 3-year study period. Among the 130 patients who reported self-stigma at baseline, 90 patients had persistent self-stigma after 1 year, 74 patients had persistent self-stigma after 2 years, and only 50 patients had persistent self-stigma after 3 years (Figure 1).

### Clinical and Demographic Features of Patients With and Without Self-Stigma

At baseline, compared with patients without self-stigma, patients with self-stigma had younger age, earlier age of onset, and higher scores of HDRS and HARS. After 3 years, compared with patients without self-stigma, patients with self-stigma had younger age, earlier age of onset, and higher scores of HDRS and HARS (Table 1).

**Table 1:** Demographic and clinical features of patients with PD.

	**Baseline**			**3-year**		
	**Without self-stigma**	**With self-stigma**	***p*-value**	**Without self-stigma**	**With self-stigma**	***p*-value**
Number of samples	94	130	/	99	96	/
Age, years, median (IQR)	60.4 (14.9)	56.5 (17.4)	0.047^*^	64.8 (15.4)	59.8 (17.2)	0.043^*^
Age of onset, years, median (IQR)	58.5 (15.2)	55.1 (18.0)	0.047^*^	59.5 (15.0)	54.9 (18.3)	0.038^*^
Disease duration, median (IQR)	1.2 (1.4)	1.6 (1.2)	0.304	5.1 (1.9)	5.3 (1.8)	0.692
Male sex, No. (%)	55 (58.5)	66 (50.8)	0.419	59 (59.6)	49 (51.0)	0.429
Education, median (IQR)	11.5 (6.0)	12.0 (6.0)	0.890	10.0 (7.0)	12.0 (6.0)	0.168
LEDD, mg/day, median (IQR)	0.0 (200.0)	75.0 (300.0)	0.304	450.0 (350.0)	450.0 (350.0)	0.692
Married, No. (%)	89 (94.7)	123 (94.6)	0.983	94 (94.9)	91 (94.8)	1.000
Work, No. (%)	25 (26.6)	46 (35.4)	0.308	27 (27.3)	34 (35.4)	0.429
Fluctuation, No. (%)	0 (0.0)	0 (0.0)	/	28 (28.3)	43 (44.8)	0.065
Dyskinesia, No. (%)	0 (0.0)	0 (0.0)	/	11 (11.1)	12 (12.5)	0.898
Antidepressant, No. (%)	1 (1.1)	7 (5.4)	0.304	10 (10.1)	15 (15.6)	0.429
FAB score, median (IQR)	17.0 (2.0)	17.0 (2.0)	0.419	17.0 (2.0)	17.0 (2.0)	0.429
MOCA score, median (IQR)	27.0 (4.0)	26.0 (4.3)	0.983	26.0 (4.0)	27.0 (4.0)	0.301
NMSS score, median (IQR)	21.0 (28.0)	25.0 (37.3)	0.304	21.0 (26.0)	22.0 (30.75)	0.692
HDRS score, median (IQR)	4.0 (7.3)	9.0 (11.7)	0.009^*^	5.0 (6.0)	8.0 (11.0)	0.019^*^
HARS score, median (IQR)	3.0 (5.0)	6.0 (9.0)	0.009^*^	5.0 (6.0)	8.0 (9.0)	0.019^*^
UPDRS II score, median (IQR)	5.5 (8.0)	6.0 (8.0)	0.492	9.0 (7.0)	9.0 (6.0)	0.429
UPDRS III score, median (IQR)	21.0 (11.2)	22.0 (17.0)	0.783	27.0 (14.0)	26.0 (11.0)	0.898
H&Y, median (IQR)	2.0 (0.5)	2.0 (0.5)	0.756	2.0 (0.5)	2.0 (0.0)	0.898

*PD, Parkinson's disease; IQR, interquartile range; LEDD, levodopa equivalent daily dose; FAB, frontal assessment battery; MOCA, Montreal Cognitive Assessment; NMSS, Non-Motor Symptoms Scale; HDRS, Hamilton Depression Rating Scale; HARS, Hamilton Anxiety Rating Scale; UPDRS, Unified Parkinson's Disease Rating Scale; H&Y, Hoehn & Yahr*.

* *Significant difference after false discovery rate (FDR) correction for multiple comparisons*.

### Associated and Predictive Factors of Self-Stigma

In the unadjusted GEE model, the variables with a *p-*value < 0.10 included age (*p* = 0.001), age of onset (*p* < 0.001), work status (*p* = 0.072), NMSS score (*p* < 0.001), HDRS score (*p* < 0.001), HARS score (*p* < 0.001), UPDRS II score (*p* < 0.001), and UPDRS III score (*p* = 0.010). Sex, antidepressant drugs (yes/no), and follow-up time in years have also been included in the adjusted model. However, most of the variables lost significance in the adjusted GEE model except for the HDRS score (B: 0.160, 95% CI: 0.106–0.214, *p* < 0.001) (Table 2).

**Table 2:** Factors associated with higher self-stigma scores in patients with PD.

	**Unadjusted model**			**Adjusted model**		
	**B**	**95% CI**	***P*-value**	**B**	**95% CI**	***P*-value**
Age	−0.056	−0.090 to −0.023	0.001^*^	0.082	−0.138 to 0.302	0.464
Age of onset	−0.068	−0.103 to −0.033	<0.001^*^	−0.152	−0.364 to 0.059	0.158
Disease duration	0.081	−0.033 to 0.194	0.164			
Female sex	0.435	−0.266 to 1.136	0.224	0.241	−0.390 to 0.872	0.454
Education	−0.011	−0.104 to 0.083	0.824			
LEDD	0.000	0.000 to 0.001	0.531			
Marriage status	−0.566	−2.409 to 1.277	0.547			
Work status	0.703	−0.064 to 1.471	0.072	−0.227	−0.971 to 0.518	0.551
Fluctuation	0.396	−0.193 to 0.986	0.188			
Dyskinesia	0.690	−0.329 to 1.709	0.184			
FAB	−0.086	−0.244 to 0.073	0.291			
MOCA	−0.048	−0.140 to 0.045	0.314			
NMSS	0.032	0.019 to 0.046	<0.001^*^	0.008	−0.007 to 0.023	0.317
HDRS	0.182	0.145 to 0.219	<0.001^*^	0.160	0.106 to 0.214	<0.001^*^
Antidepressant	0.412	−0.619 to 1.443	0.434	0.320	−0.603 to 1.243	0.497
HARS	0.161	0.116 to 0.205	<0.001^*^	0.002	−0.069 to 0.072	0.959
UPDRS II	0.087	0.040 to 0.133	<0.001^*^	0.044	−0.005 to 0.092	0.076
UPDRS III	0.031	0.007 to 0.054	0.010^*^	−0.011	−0.037 to 0.015	0.397
H&Y	0.430	−0.075 to 0.935	0.095			
Follow-up time in years	0.074	−0.100 to 0.247	0.405	−0.059	−0.403 to 0.285	0.738

*PD, Parkinson's disease; LEDD, levodopa equivalent daily dose; FAB, frontal assessment battery; MOCA, montreal cognitive assessment; NMSS, non-motor symptoms scale; HDRS, hamilton depression rating scale; HARS, hamilton anxiety rating scale; UPDRS, unified parkinson's disease rating scale; H&Y, Hoehn & Yahr*.

* *Significant difference*.

A total of 94 patients who had no self-stigma at baseline were included in the analysis for the detection of predictive factors of self-stigma. Six variables have been included in the multivariate logistic model, including age, sex, age of onset, and scores of HDRS, HARS, and UPDRS III. Only HDRS scores remained in the multivariate logistic analysis (*p* = 0.015) (Table 3).

**Table 3:** Predictive factors for the development of self-stigma in patients with PD (*n* = 94).

	**Unadjusted model**			**Adjusted model**		
	**OR**	**95% CI**	***P*-value**	**OR**	**95% CI**	***P*-value**
Age	0.940	0.898–0.984	0.008^*^	1.502	0.875–2.578	0.140
Female sex	1.174	0.516–2.669	0.702	1.214	0.474–3.107	0.686
Age of onset	0.937	0.894–0.981	0.006^*^	0.615	0.357–1.061	0.081
Disease duration	1.336	0.841–2.121	0.220			
Education	0.980	0.881–1.090	0.707			
Marriage status	4.091	0.440–38.059	0.216			
Work status	1.182	0.473–2.953	0.721			
LEDD	1.000	0.997–1.002	0.773			
FAB	1.151	0.884–1.499	0.295			
MOCA	1.034	0.880–1.214	0.688			
NMSS	0.986	0.963–1.010	0.246			
HDRS	1.073	0.986–1.168	0.104	1.252	1.044–1.502	0.015^*^
Antidepressant	0.000	/	1.000			
HARS	1.035	0.946–1.133	0.452	0.909	0.753–1.098	0.324
UPDRS II	0.991	0.902–1.090	0.857			
UPDRS III	0.972	0.927–1.018	0.231	0.949	0.897–1.005	0.075
H&Y	0.881	0.377–2.056	0.769			

*PD, Parkinson's disease; LEDD, levodopa equivalent daily dose; FAB, frontal assessment battery; MOCA, montreal cognitive assessment; NMSS, non-motor symptoms scale; HDRS, hamilton depression rating scale; HARS, hamilton anxiety rating Scale; UPDRS, unified parkinson's disease rating scale; H&Y, Hoehn & Yahr*.

* *Significant difference*.

### Self-Stigma and Depression

Considering the results that the HDRS score was the only associated and predictive factor of self-stigma in patients with PD, we conducted further analyses to explore the association between self-stigma and depression. As shown in Figure 2A, the prevalence of self-stigma had a decreasing tendency, the frequency of antidepressant drug use had an increasing tendency, and the prevalence of depression remained relatively stable during follow-up. Then, we divided the included patients into depression and non-depression groups with a cutoff value of 20 of the HDRS-24. Both the depression and non-depression groups showed a decreasing prevalence of self-stigma over 3 years (Figure 2C). The relative risk (RR) for self-stigma was stable (range: 1.4–1.6) between the depression and non-depression groups over 3 years. Moreover, the scores of UPDRS II, UPDRS III, axial symptoms, and facial symptoms showed a tendency of increasing over 3 years (Figure 2B).

## Discussion

The current longitudinal cohort study showed that the prevalence of self-stigma in PD was 58.0% at baseline and 52.2, 56.3, and 49.2% at 1-, 2-, and 3-year follow-ups. The prevalence was consistent with a previous study, which showed about half of the patients with PD had self-stigma (Ma et al., 2016). The results indicated that self-stigma was frequent in patients with PD, especially at a very early stage of the disease.

Although several studies have explored the associated factors of self-stigma in PD, all of them were of cross-sectional design and lacked longitudinal data. The results were also inconsistent: depression has been reported to correlate with self-stigma in four studies (Schrag et al., 2001; Oguru et al., 2010; Ma et al., 2016; Salazar et al., 2019) but not in another three studies (Chapuis et al., 2005; Wu et al., 2014; da Silva et al., 2020). Younger age has been reported to correlate with self-stigma in two studies (Wu et al., 2014; Salazar et al., 2019) but not in another five studies (Schrag et al., 2001; Chapuis et al., 2005; Oguru et al., 2010; Ma et al., 2016; da Silva et al., 2020), while apathy, difficulties in ADL, non-motor symptoms, H&Y stage, marital status, biphasic dyskinesia, morning akinesia, end-of-dose fluctuations, and unpredictable offs have been reported to correlate with self-stigma only in a single study (Chapuis et al., 2005; Oguru et al., 2010; Wu et al., 2014; da Silva et al., 2020). The heterogeneous results might partially ascribe to the different cultural backgrounds as self-stigma originates in the social context (Major and O'Brien, 2005). By using the GEE model, which allowed for correlation between repeated measurements of the same patients, we identified that HDRS score was the only factor associated with self-stigma in the Chinese population.

No prospective study has been conducted before to investigate the risk factors for self-stigma. We also found that only the HDRS score could predict the later development of self-stigma during the 3 years of follow-up.

The role of depression in the development of self-stigma in PD observed in this study was in accordance with the previous cross-sectional studies (Schrag et al., 2001; Oguru et al., 2010; Ma et al., 2016; Salazar et al., 2019). In addition, just as self-stigma, depression has also been widely reported to correlate with poor quality of life and high burden for caregivers (Slawek et al., 2005; Qin et al., 2009; Genc et al., 2019).

In a previous study, patients with PD with higher facial masking were perceived by health practitioners to be more depressed, less sociable, and less socially supportive than those with low levels of facial masking (Tickle-Degnen et al., 2011), which support the hypothesis that motor symptoms such as facial symptoms might fuel negative social perceptions and lead to consecutive self-stigma. Previous studies have reported a reduction in self-stigma after deep brain stimulation (DBS) surgery, also suggesting a potential relationship between self-stigma and motor severity (Lyons and Pahwa, 2005; Ellis et al., 2008). However, we failed to detect any relationship between motor symptoms and self-stigma. With the progression of the disease, the motor symptoms deteriorated, but the self-stigma became less frequent on the contrary (Figures 2A,B).

A previous study has found that the impact of motor symptoms and difficulties in ADL on self-stigma was completely mediated by depression (Salazar et al., 2019), namely, only depression was the real trigger of the internalization of social stigma. This might be a rational explanation of our findings. Thus, treatments only targeted at alleviating motor symptoms might not alleviate stigma perception since only depression was the true contributor of self-stigma. More visibly, as shown in Figures 2A,C, the prevalence of self-stigma was inversely correlated with the frequency of antidepressant drug use, and the RR for self-stigma nearly doubled in patients with PD with depression compared with patients with PD without depression, also indicating that depression could be a good target to deal with self-stigma in patients with PD.

Our study revealed the evolution of self-stigma as a non-persistent and overall decreasing symptom for the first time. These findings implicated that self-stigma was reversible, so more attention should be paid to self-stigma in clinical practice. We noticed that the prevalence of depression remained relatively stable during follow-up (Figure 2A), which was in line with a previous study (Zhu et al., 2016). Besides, after dividing the included patients into depression and non-depression groups, both groups showed a decreasing tendency of self-stigma over 3 years (Figure 2C). Therefore, the decreasing tendency of self-stigma detected in this study could not ascribe to a decrease in depression; rather, it might be an intrinsic characteristic of self-stigma in PD. An acceptance of the disease with time duration or proper patient education might play a role.

This study has some strengths. First, it was the first prospective cohort study focusing on self-stigma in PD, so that we could observe the evolution of self-stigma in a longitudinal cohort. Second, this study analyzed repeated measurement data using the GEE model, which can minimize the loss of information. Third, we conducted a comprehensive analysis of the determinants of self-stigma by including nearly all variables that have been reported to correlate with self-stigma in PD (Hanff et al., 2021), such as age, sex, educational attainment, marriage status, anxiety, depression, non-motor symptoms, motor symptoms, and motor complications. Fourth, all of the included patients were of Han nationality, which avoids the cultural heterogeneity.

However, several limitations should also be acknowledged. First, self-stigma was measured by four items of the PDQ-39 rather than a specialized scale. And we defined the presence of self-stigma as a total stigma score ≥ 1 of 16, which might have overstated the prevalence of self-stigma. Second, the follow-up time was relatively short and the included patients were at an early stage with relatively mild motor symptoms, which might preclude consideration of potentially stigma-inducing assistive devices such as canes and walkers. Longer follow-up is required to further confirm the current findings. Third, there were still some variables that may influence self-stigma that were not included, such as apathy, psychiatric disease, and personality of the patients.

## Conclusion

Self-stigma is very common in PD, but its prevalence tends to decrease as the disease progresses. Depression was the only associated risk factor of self-stigma in PD and could be an effective target of improving self-stigma.

## Data Availability Statement

The original contributions presented in the study are included in the article/Supplementary Material, further inquiries can be directed to the corresponding author/s.

## Ethics Statement

The studies involving human participants were reviewed and approved by Ethics Committee of West China Hospital of Sichuan University. The patients/participants provided their written informed consent to participate in this study.

## Author Contributions

JL contributed to conception, organization and execution, data collection and statistical analysis, and drafting the manuscript. RO contributed to execution, data collection, and statistical analysis. QW, BC, YH, LZ, and KL contributed to execution and data collection. CL contributed to conception, organization, execution, and data collection. HS contributed to conception and organization, manuscript review and critique, and was responsible for the overall content as the guarantor. All authors contributed to the article and approved the submitted version.

## Funding

This study was supported by the National Key Research and Development Program of China (Grant No. 2016YFC0901504), the 1.3.5 project for disciplines of excellence, West China Hospital, Sichuan University (Grant No. ZYJC18038), the Science Foundation of Chengdu Science and Technology Bureau (Grant No. 2019-YF05-00307-SN), and the funding of the National Science Fund of China (Grant No. 81901293).

## Conflict of Interest

The authors declare that the research was conducted in the absence of any commercial or financial relationships that could be construed as a potential conflict of interest.

## Publisher's Note

All claims expressed in this article are solely those of the authors and do not necessarily represent those of their affiliated organizations, or those of the publisher, the editors and the reviewers. Any product that may be evaluated in this article, or claim that may be made by its manufacturer, is not guaranteed or endorsed by the publisher.
