# Supplementary material for: Self-Stigma in Parkinson's Disease: A 3-Year Prospective Cohort Study
Source: Front Aging Neurosci. 2022 Feb 11;14:790897. doi: 10.3389/fnagi.2022.790897 (PMC8877567; doi:10.3389/fnagi.2022.790897)
Supplement: Supplementary file 1 [file Table_1.pdf]

Supplementary Table 1 Measurements of the clinical characteristics of the included PD patients

Characteristics	Assessment Scales	Assessment time points
Motor symptoms	UPDRS III	Baseline and 1,2,3-year follow-up
Non-motor symptoms	NMSS	Baseline and 1,2,3-year follow-up
Activities of daily living	UPDRS II	Baseline and 1,2,3-year follow-up
Disease stage	H&Y	Baseline and 1,2,3-year follow-up
Depression	HDRS	Baseline and 1,2,3-year follow-up
Anxiety	HARS	Baseline and 1,2,3-year follow-up
Global cognitive function	MOCA	Baseline and 1,2,3-year follow-up
Executive function	FAB	Baseline and 1,2,3-year follow-up
Fluctuation	UPDRS IV	Baseline and 1,2,3-year follow-up
Dyskinesia	UPDRS IV	Baseline and 1,2,3-year follow-up
Self-tigma	Stigma subscale of the PDQ-39	Baseline and 1,2,3-year follow-up

Abbreviations: PD, Parkinson's disease; UPDRS, Unified Parkinson's Disease Rating Scale; NMSS, Non-Motor Symptoms Scale; H&Y, Hoehn & Yahr; HDRS, Hamilton Depression Rating Scale; HARS, Hamilton Anxiety Rating Scale; MOCA, Montreal Cognitive Assessment; FAB, frontal assessment battery; PDQ-39, Parkinson's Disease Questionnaire-39.

Supplementary Table 2 Demographic and clinical features of PD patients with and without 3-year follow-up completed

	3-year follow-up completed	3-year follow-up not completed	p value
Number of samples	195	29	/
Age, years, median (IQR)	59.4 (17.4)	54.5 (13.3)	0.570
Age of onset, years, median (IQR)	58.0 (17.8)	52.8 (13.6)	0.570
Disease duration, median (IQR)	1.5 (1.3)	1.6 (1.0)	0.795
Male sex, No. (%)	108 (55.4)	13 (44.8)	0.795
Education, median (IQR)	12.0 (6.0)	10.0 (6.0)	0.795
LEDD, mg/day, median (IQR)	0.0 (300.0)	0.0 (225.0)	0.795
Married, No. (%)	185 (94.9)	27 (93.1)	0.795
Work, No. (%)	61 (31.3)	10 (34.5)	0.795
Self-stigma, No. (%)	112 (57.4)	18 (62.1)	0.795
Self-stigma score, median (IQR)	1.0 (4.0)	2.0 (5.5)	0.795
Antidepressant, No. (%)	8 (4.1)	0 (0.0)	0.795
FAB score, median (IQR)	17.0 (2.0)	17.0 (2.5)	0.795
MOCA score, median (IQR)	27.0 (4.0)	26.0 (4.5)	0.795
NMSS score, median (IQR)	22.0 (34.0)	22.0 (31.5)	0.795
HDRS score, median (IQR)	6.0 (10.0)	6.0 (10.0)	0.795
HARS score, median (IQR)	5.0 (7.0)	4.0 (8.5)	0.795
UPDRS II score, median (IQR)	6.0 (8.0)	5.0 (8.0)	0.795

UPDRS III score, median (IQR)	22.0 (14.0)	20.0 (14.5)	0.795
H&Y, median (IQR)	2.0 (0.5)	2.0 (1.0)	0.795

Abbreviations: PD, Parkinson's disease; IQR, interquartile range; LEDD, levodopa equivalent daily dose; FAB, frontal assessment battery; MOCA, Montreal Cognitive Assessment; NMSS, Non-Motor Symptoms Scale; HDRS, Hamilton Depression Rating Scale; HARS, Hamilton Anxiety Rating Scale; UPDRS, Unified Parkinson's Disease Rating Scale; H&Y, Hoehn & Yahr.

* Significant difference after false discovery rate (FDR) correction for multiple comparisons.
